# Molecular Phylogeny, Divergence Time Estimation, and Biogeography of *Moelleriella* (Clavicipitaceae, Hypocreales) with Taxonomic Insights

**DOI:** 10.3390/biology15100739

**Published:** 2026-05-07

**Authors:** Yongsheng Lin, Jiao Yang, Nemat O. Keyhani, Luxiao Wang, Yuhang Yao, Xiuyan Wei, Feifei Song, Zhenxing Qiu, Shouping Cai, Xiayu Guan, Lin Zhao, Junzhi Qiu

**Affiliations:** 1 State Key Laboratory of Agricultural and Forestry Biosecurity, College of Life Sciences, Fujian Agriculture and Forestry University, Fuzhou 350002, China; linyongsheng0909@163.com (Y.L.); jiaoyang0368@163.com (J.Y.); wanglx1001@163.com (L.W.); y312960@163.com (Y.Y.); xiuyanwei@yeah.net (X.W.); flyinthesky-feifei@163.com (F.S.); 2 Department of Biological Sciences, University of Illinois, Chicago, IL 60607, USA; keyhani@uic.edu; 3 College of Humanities and Law, Fuzhou Technology and Business University, Fuzhou 350715, China; qiuzhenxing2006@126.com; 4 Fujian Academy of Forestry, Fuzhou 350012, China; caishouping@163.com; 5 Key Laboratory of Ministry of Education for Genetics, Breeding and Multiple Utilization of Crops, College of Horticulture, Fujian Agriculture and Forestry University, Fuzhou 350002, China; gxy302@126.com

**Keywords:** Clavicipitaceae, ancestral state reconstruction, molecular clock, multi-locus phylogeny, fungal taxonomy

## Abstract

The Clavicipitaceae family, including saprobes and insect and myco-pathogens, are widely distributed in nature across various trophic regions, and play important roles in insect population control, plant interactions, and symbiotic evolution. Members of the genus *Moelleriella* within this family primarily specialize in infecting scale insects and whiteflies. Using five genomic loci (SSU, LSU, *tef1-α*, *rpb1*, and *rpb2*), we report on the inferred divergence times among members of Clavicipitaceae using molecular dating analyses. Molecular clock estimates revealed that the ancestor of *Moelleriella* likely emerged in the Late Cretaceous (91.60 Mya; 95% highest posterior density of 79.29–100.13 Mya). Historical biogeographic reconstruction of *Moelleriella*, performed using the Bayesian Binary Markov chain Monte Carlo (BBM) method, indicates that it most likely originated in Asia. Moreover, based on taxonomic and phylogenetic analyses, we describe three species within the genus *Moelleriella*, including one new species (*Moelleriella microstroma*) and two new records for China (*Moelleriella chiangmaiensis* and *Moelleriella phukhiaoensis*).

## Simple Summary

This study describes one new species and two new Chinese records within the insect pathogenic genus, *Moelleriella*. Ancestral-area reconstruction suggests an Asian origin for *Moelleriella* during the Late Cretaceous (91.60 Mya), with subsequent dispersal to North America and South America via the Bering land bridge and to Africa via the Arabian Peninsula land bridge. These findings provide a phylogenetic framework for understanding the origin and evolutionary history of this genus.

## 1. Introduction

The genus *Moelleriella* Bres. (Ascomycota, Hypocreales, Clavicipitaceae) comprises entomopathogenic fungi that parasitize scale insects (Coccidae, Lecaniidae) and whiteflies (Aleyrodidae) [1]. These fungi are predominantly distributed in tropical ecosystems and are occasionally found in subtropical regions [2,3]. Originally classified under *Hypocrella* Sacc., the genus was subsequently segregated based on molecular phylogenetic analyses and morphological revisions from allied genera (e.g., *Samuelsia* P. Chaverri & K.T. Hodge, *Hypocrella* P.A. Saccardo, and *Orbiocrella* D. Johnson, G.H. Sung, Hywel-Jones & Spatafora) [2,4]. *Moelleriella sulphurea* Bres. was designated as the type species of the genus [5]. *Moelleriella* differs from *Samuelsia* and *Hypocrella* in that its ascospores are fibrillar, multiseptate, and disarticulate at the septa within the ascus, whereas those of *Samuelsia* and *Hypocrella* remain intact, appearing filiform or long fusiform [1,6]. Furthermore, the anamorph of *Moelleriella* produces fusiform conidia, contrasting sharply with the allantoid conidia of *Samuelsia* [7,8]. Another notable characteristic of *Moelleriella* species is their brightly colored and morphologically diverse stromata, which often exhibit vivid hues of yellow, orange, or white [6]. Stromatal structures range from globose, thick pulvinate, tuberculate, and convex-to-thin pulvinate, serving as key diagnostic traits for species identification [9]. These morphological distinctions, combined with molecular evidence from loci such as the large subunit of ribosomal RNA (LSU) gene, the translation elongation factor 1-α (*tef1-α*) gene, and the largest subunit of RNA polymerase II (*rpb1*), support the taxonomic distinction of this genus [2].

These fungi exhibit dual trophic association strategies: necrotrophic parasitism on insects and biotrophic interactions with host plants, which combine to significantly enhance their survival and dispersal in complex environments [3,10]. According to the Index Fungorum database (https://indexfungorum.org/, accessed on 26 January 2026), *Moelleriella* currently comprises 83 species, with transcontinental distributions in some species (e.g., *Moelleriella libera,* found in Bolivia, Panama and regions of China; *Moelleriella ochracea* in Honduras and Brazil; and *Moelleriella raciborskii* in Ghana and Thailand) suggesting historical dispersal or vicariance events [2]. Such dispersals can be addressed via biogeographic studies by reconstructing the origins, speciation, and distribution patterns of organisms (fungi). Hyde et al. proposed a series of evolutionary timeframes [11], which indicated that fungal phyla originated >550 Mya, subphyla of Ascomycota emerged ~400–550 Mya, classes ~300–400 Mya, and subclasses ~250–300 Mya. Through molecular dating and ancestral reconstruction, the ancestor of the Schizoparmaceae family within the Diaporthales (sac fungi, includes several important plant pathogens and saprobes) was inferred to have likely originated in Africa during the Late Cretaceous, approximately 75.7 Mya (60.3–91.3 Mya) [12]. Similarly, investigations of the fungal spider pathogens, *Akanthomyces sensu lato* within the family Cordycipitaceae, suggests that the ancestor of this group appeared in the Paleogene, around 34.57 Mya (31.41–37.67 Mya), most likely originating in Asia [13]. Thailand and China have emerged as biodiversity hotspots for *Moelleriella*, with recent studies reporting thirteen new species from Yunnan, Hainan and Sichuan provinces in China [3,9,14], suggesting a continued biodiversity of the genus, and providing a valuable dataset for biogeographic studies.

Here, we isolated and purified specimens collected from Guizhou, Hunan, and Jiangxi provinces in China, and analyzed nucleotide sequences from three genetic loci (LSU, *tef1-α* and *rpb1*). Based on morphological characteristics and molecular data, we describe one new species and two new records in China, providing detailed illustrations and taxonomic descriptions. Furthermore, using *Paleoophiocordyceps coccophagus* (G.H. Sung, Poinar & Spatafora) as a fossil calibration point, we estimated the divergence time and origin of the genus *Moelleriella*, thereby offering new insights into its evolutionary history, biogeography, and likely routes of dispersal.

## 2. Materials and Methods

### 2.1. Collections and Isolation

*Moelleriella* specimens were collected from the undersides of dicotyledonous plant leaves at the following three locations in China: (1) Maolan Reserve, Libo County, Guizhou Province (107°52′10″–108°45′40″ E, 25°09′20″–25°20′50″ N); (2) Taoyuandong Reserve, Yanling County, Hunan Province (113°56′30″–114°06′20″ E, 26°18′00″–26°35′30″ N); and (3) Jiulianshan Reserve, Longnan County, Jiangxi Province (114°30′–114°50′ E, 24°30′–24°50′ N). Specimens were placed into 50 mL tubes and brought to the laboratory for isolation and purification by tissue isolation. Specimens collected in the field were first washed with sterile water on a sterile workbench, and using sterilized forceps and a scalpel, a portion of the stroma was excised and immersed in 75% ethanol (Sinopharm Chemical Reagent Co., Ltd., Shanghai, China) for 30 s, followed by rinsing in ddH_2_O for 60 s. After drying on sterile filter paper, the stroma was placed in a sterile Petri dish, and 200 μL of ddH_2_O was added. The stroma was then gently crushed with the handle of a sterilized scalpel, and the resulting suspension was spread onto potato dextrose agar (PDA) plates supplemented with 10 mg/mL ampicillin (Solarbio Science and Technology Co., Ltd., Beijing, China). After incubation at 25 °C for 3–5 d, the growing edge of a single fungal colony was transferred to a fresh PDA plate to obtain a pure culture. Dried specimens and purified isolates were deposited in the Mycological Herbarium, Institute of Mycology, Chinese Academy of Sciences (HMAS), and the China General Microbiological Culture Collection Center (CGMCC).

### 2.2. Morphological Observations

Fresh specimens were photographed in the field using a Canon EOS 6D Mark II camera (Canon Inc., Tokyo, Japan) for preliminary morphological documentation. Macroscopic features were examined and photographed in the laboratory under a stereomicroscope (Nikon SMZ74, Tokyo, Japan). After fungal pure cultures were inoculated onto PDA plates and incubated at 25 °C for 21 d, colony photographs were taken using the same Canon camera, and the resultant images used to measure colony diameters [5]. Tissue sections of specimens were mounted on glass slides with lactic acid–cotton blue (Solarbio Science and Technology Co., Ltd., Beijing, China), and microscopic structures were observed and photographed using a Nikon Ni-U compound microscope (Ni-U, Tokyo, Japan) [15]. All images were analyzed using Digimizer software (v5.4.4). The lengths and widths of thirty conidia and pycnidia were measured/sampled.

### 2.3. DNA Extraction and PCR Amplification

Small pieces of tissue were taken from the PDA plates of each sample, crushed in a mortar with liquid nitrogen, and total genomic DNA was extracted using the Fungal DNA Mini Kit (OMEGA–D3390, Feiyang Biological Engineering Corporation, Guangzhou, China) following the manufacturer’s instructions. The concentration and purity of total DNA were measured using a Nano-400A Ultramicro nucleic acid analyzer (AllSheng Company, Hangzhou, China). For molecular/phylogenetic determination, the sequences of the nuclear genomic loci were examined: (i) the nuclear ribosomal large subunit (LSU), (ii) translation elongation factor1-α gene (*tef1-α*), and (iii) RNA polymerase II largest subunits (*rpb*1) [2,16]. The primers and thermocycler conditions for PCR amplification are given in Table 1. Using the 2 × Rapid Taq Master Mix kit (Vazyme, Nanjing, China), target genes were amplified via polymerase chain reaction (PCR) using a thermal cycler (Bio-Rad, Hercules, CA, USA). For each sample, a 25 μL reaction volume included 12.5 μL of 2 × Rapid Taq Master Mix, 1 μL each of forward and reverse primers (10 μM) (QingKe Biotech Co., Ltd., Beijing, China), 1 μL of genomic DNA (100–200 ng/μL), and 9.5 μL of sterile water. The PCR products underwent purification and Sanger sequencing by BioSune (BioSune Co., Ltd., Shanghai, China). New sequences generated in this study have been deposited in GenBank (https://www.ncbi.nlm.nih.gov/, accessed on 28 January 2026) with accession numbers given in Table 2.

### 2.4. Sequence Alignment and Phylogenetic Analyses

Based on the Sanger sequencing results, the sequences of the LSU, *tef1-α*, and *rpb1* genes were manually adjusted using MEGA v.7.0 and BioEdit v.7.2.6.1 [22,23]. The adjusted sequences were subjected to a BLAST (https://blast.ncbi.nlm.nih.gov/Blast.cgi/, accessed on 28 January 2026) search via the NCBI (National Center for Biotechnology Information). In combination with the reported literature, GenBank accession numbers for phylogenetic analysis were downloaded from the NCBI (Table 2). MAFFT v.7.11 (https://mafft.cbrc.jp/alignment/software/, accessed on 28 January 2026) was used to align each locus individually, and the primer-derived sequences were then trimmed using MEGA 7.0 [24]. The trimmed sequences were concatenated using Phylosuite v1.2 [25]. Phylogenetic analyses were performed on the concatenated three-gene dataset using Maximum Likelihood (ML) and Bayesian Inference (BI). ML was conducted in IQtree 1.6.8 with automatic model selection and 1000 bootstrap iterations. BI was performed using MrBayes 3.2.6. The best-fit evolutionary model for each partition was selected under the Akaike Information Criterion (AIC) using PartitionFinder 2 [26]. Four parallel Markov chain Monte Carlo (MCMC) simulations were run for 2,000,000 generations with a sampling frequency of every 100th generation. The burn-in phase was set at 10% of the total runs [27]. Finally, the phylogenetic trees were visualized using FigTree v.1.4.3 and edited with Adobe Illustrator CS 6.0 (Adobe Systems Inc., San Jose, CA, USA).

### 2.5. Divergence Time Estimation and Inferring Historical Biogeography

The divergence times of *Moelleriella* species were inferred using the Bayes MCMC algorithm in the BEAST 2.7.5 software package. The analysis was based on the SSU + LSU + *tef1-α* + *rpb1*+ *rpb2* concatenated sequence dataset from 159 specimens [28]. MrModeltestv.2.3 was used to select the best-fitting evolutionary model (GTR + G + I) for the dataset. The Extensible Markup Language (XML) file was imported into BEAUti v2.0 for parameter configuration. A relaxed clock log normal model was applied [29,30]. Fossil calibration was performed using *P. cocophagus*, a fungal parasite of a scale insect from the Cretaceous period (99–105 Mya) [31]. A gamma-distributed prior was applied for fossil node calibration. This calibration was used to estimate the molecular clock ages of the nodes for species within the family Clavicipitaceae. The BEAST analysis ran for 200 million generations, logging parameters every 10,000 generations. Convergence and stationarity of the resulting log files were checked using Tracer v.1.7 soft, with ESS values ≥ 200 indicating convergence [12]. The first 10% of trees, representing the initial and unreliable results, were discarded as burn-in. Finally, a maximum clade credibility tree was constructed using TreeAnnotator v. 2.6.7 [13,32].

To reconstruct the ancestral geographical distribution and infer the historical biogeography of *Moelleriella*, the Bayesian Binary Markov chain Monte Carlo (BBM) method in the Reconstruct Ancestral State in Phylogenies (RASP v.4.3 software) package was used for analysis [33,34]. The parameters were set to 10 million generations, and the first 10% of samples were discarded as burn-in. Statistically, the geographic distributions of *Moelleriella* were identified in four areas: (A) Asia, (B) Africa, (C) North America, and (D) South America.

## 3. Results

### 3.1. Phylogenetic Analyses

**Figure 1:**
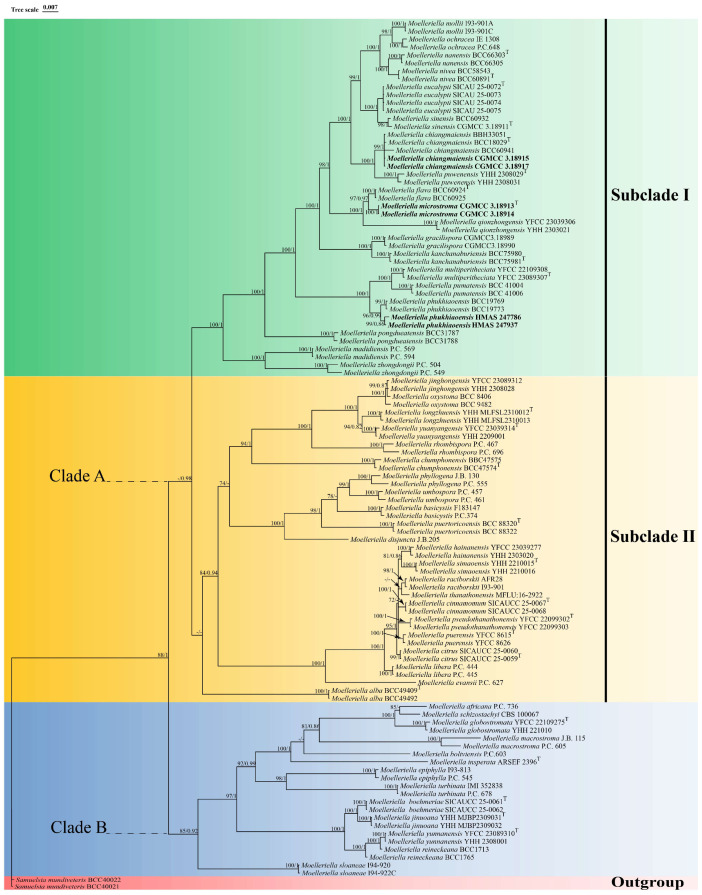
Phylogenetic analysis of *Moelleriella* based on Maximum Likelihood (ML) and Bayesian Inference (BI) using three-gene combination (LSU, *tef1-α*, and *rpb1*). The values of the BI posterior probability (≥0.70) and ML bootstrap proportions (≥70%) are indicated at the nodes (BP/PP). Fungal isolates from this study are shown in bold. The ex-type, ex-epitype, ex-neotype strains are marked with “^T^”.

Phylogenetic analyses based on ML (Maximum Likelihood) and BI (Bayesian Inference) were conducted on a dataset of 108 concatenated nuclear gene sequences (LSU + *tef1-α* + *rpb1*) from 54 species of *Moelleriella*, with *Samuelsia mundiveteris* BCC 40021 and *Samuelsia mundiveteris* BCC 40022 designated as outgroups. The concatenated three-gene sequence dataset had a total length of 2468 bp, including 869 bp for LSU, 886 bp for *tef1-α* and 713 bp for *rpb1*. The topologies inferred from the ML and BI phylogenetic analyses were largely consistent across most branches, with strong support for most branches (Figure 1). The results show that *Moelleriella* species can be divided into two distinct clades (Clade A and Clade B), with Clade A further split into two sister subclades (Subclade I and Subclade II). Sequences generated from a new species described in the present study (*Moelleriella microstroma*) and two new records of species (*Moelleriella chaiangmaiensis* and *Moelleriella phukhiaoensis*) were included in the analyses (two isolates of each, six sets of sequences total) and all were found to be located within Subclade I of Clade A. Molecular analyses show that the newly described species *M. microstroma* sp. nov. is closely related to *M. flava*, with high levels of support (BP = 97%, PP = 0.97). Two samples formed a monophyletic clade with *M. chiangmaiensis*, which was recently reported in Thailand (BP = 99%, PP = 1), while two other samples were closely related to *M. phukhiaoensis* (BP = 96%, PP = 0.99).

### 3.2. The Divergence Time Estimation of Moelleriella

**Figure 2:**
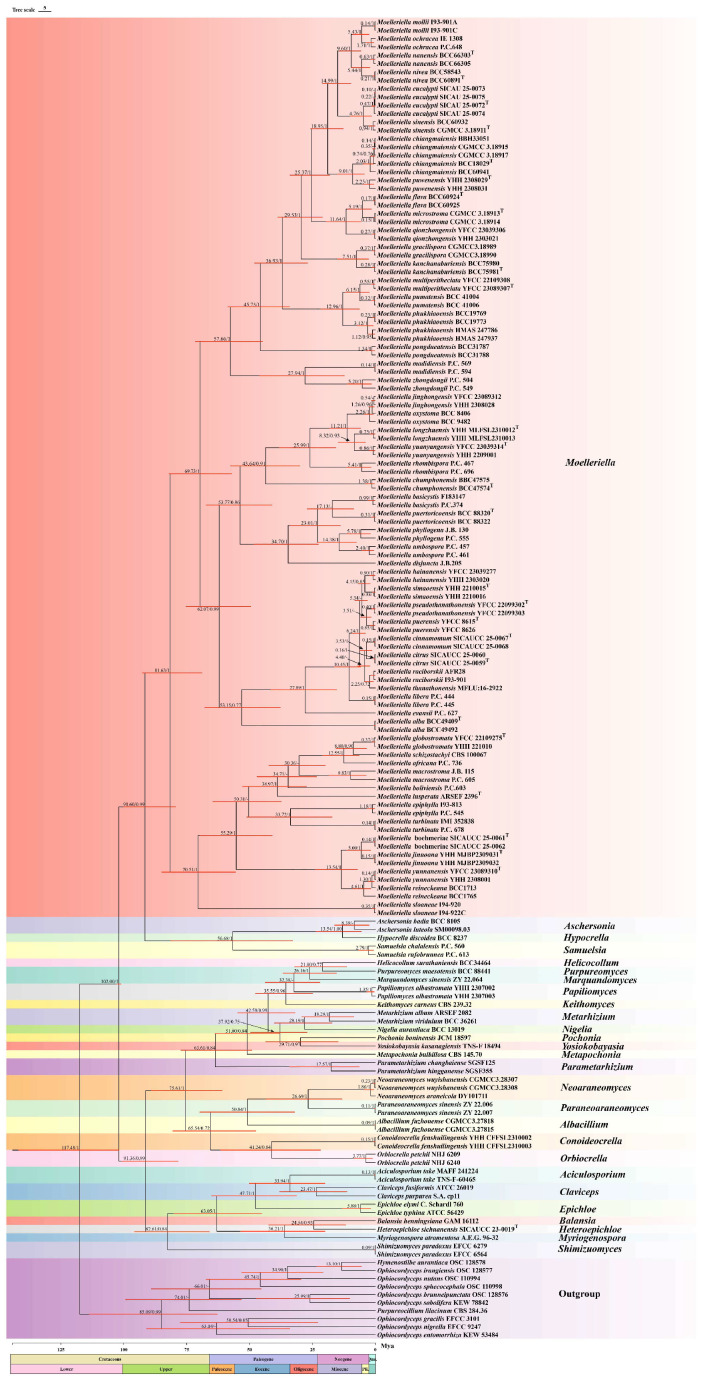
Divergence time estimates of Clavicipitaceae inferred from molecular clock analyses based on a five-locus dataset (SSU, LSU, *tef1-α*, *rpb1*, *rpb 2*). Horizontal red bars at nodes represent 95% highest posterior density (HPD) intervals for estimated divergence times. Mean divergence ages and Bayesian posterior probabilities (BPP) ≥ 0.70 are indicated at each node. Time scale in millions of years (Mya). The ex-type, ex-epitype, ex-neotype strains are marked with “^T^”.

Based on prior studies, additional members of the family Clavicipitaceae were selected to investigate the evolutionary history, origin, and internal systematics of *Moelleriella* [19,35]. Divergence time estimation analyses confirmed that Clavicipitaceae diverged during the Early Cretaceous period (Figure 2). Further analyses indicated that the divergence time of *Moelleriella* from other genera of Clavicipitaceae is closely associated, with a mean stem age of 91.60 Mya (95% HPD = 79.29–100.13 Mya; PP = 0.99) and a mean crown age of 81.63 Mya (95% HPD = 68.88–92.68 Mya; PP = 1.00). The initial diversification of *Moelleriella* occurred in the Upper Cretaceous period (66–100.5 Mya), whereas the divergence of most *Moelleriella* species took place primarily during the Neogene period (2.58–23.03 Mya).

### 3.3. The Historical Biogeography of Moelleriella

**Figure 3:**
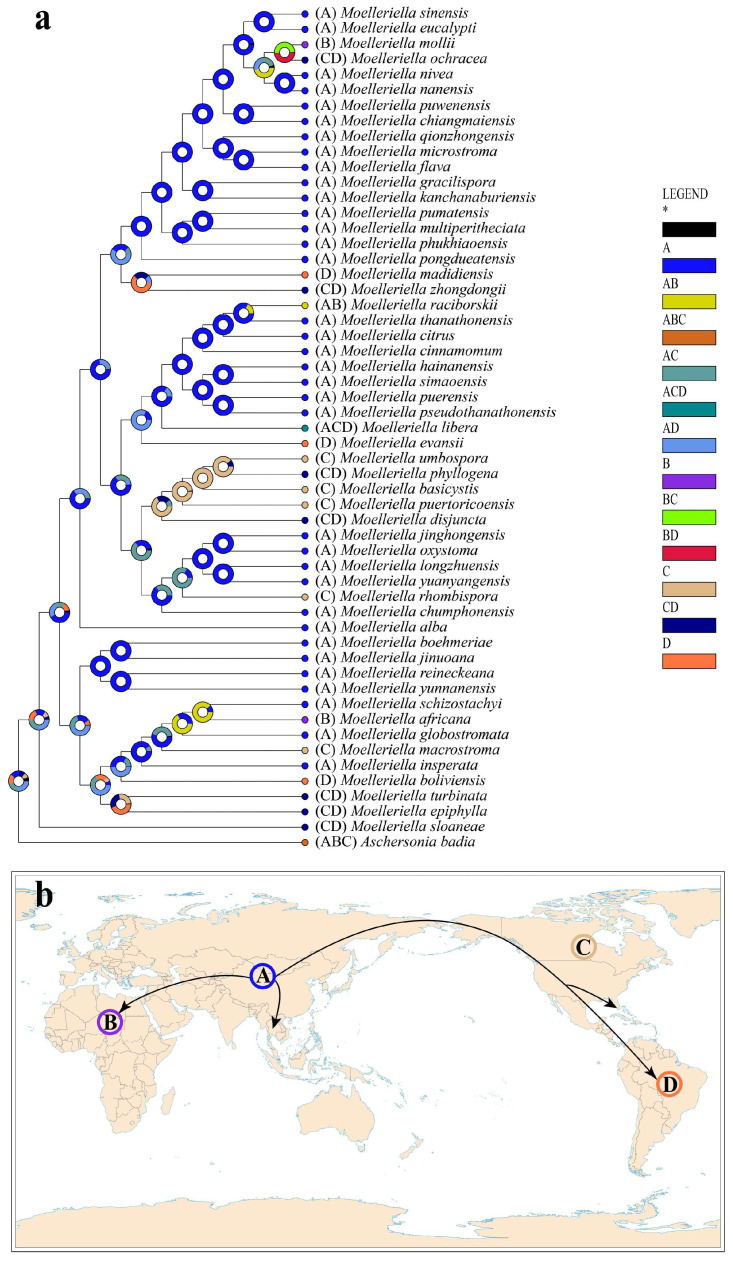
Ancestral state reconstruction and divergence time estimates of *Moelleriella*. (**a**) Pie charts at nodes represent inferred ancestral distributions from Bayesian Binary Markov chain Monte Carlo (BBM) analysis implemented in RASP. “*” represents other ancestral ranges. (**b**) Putative dispersal routes of *Moelleriella*.

Historical biogeographic scenarios of *Moelleriella* were reconstructed using RASP (Figure 3). Results from Bayesian Binary Markov chain Monte Carlo (BBM) analysis suggest an Asian origin for *Moelleriella*, with at least 25 dispersal events and 10 vicariance events shaping its current distribution. To date, 37 species have been recorded in Asia, 13 in North America, 11 in South America, and three in Africa, confirming Asia as the center of diversity for this genus.

### 3.4. Taxonomy

#### 3.4.1. *Moelleriella microstroma* X. Y. Wei, Y. S. Lin and J. Z. Qiu, sp. nov. (Figure 4)

**MycoBank.** MB861130.

**Etymology.** *Microstroma* refers to the small macroscopic morphological characteristics of the stroma.

**Diagnosis.** *Moelleriella microstroma* stroma color and conidial morphology are like those of *Moelleriella flava*, but *M. microstroma* produces multiple conidiomata on the stroma, whereas *M*. *flava* possesses only a single conidioma.

**Type.** China. Hunan Province: Zhuzhou City, Yanling County, Taoyuandong National Nature Reserve, 113°56′30″–114°06′20″ E, 26°18′00″–26°35′30″ N, collected from the abaxial surface of dicot leaves. 15 October 2017; coll. X. Y. Wei and J. Z. Qiu (holotype HMAS 247794, ex-type living culture CGMCC 3.18913).

**Description.** Anamorph stromata when immature pale yellow, subglobose, becoming nearly cylindrical with a depressed apex at maturity, 1.18 (0.78–1.64) × 1.03 (0.56–1.59) mm in diameter, and 0.51 (0.39–0.73) mm high; with a pale yellow hypothallus and multiple orange acervular conidiomata; hypothallus 0.31 (0.13–0.60) mm wide. Multiple conidiomata per stroma; conidiomatal chambers opening irregularly, arranged on both sides of the stroma. Conidia fusoid, slightly curved on one side, 11.42 (10.09–13.07) × 1.99 (1.51–2.40) μm, L/W ratio = 5.8.

**Teleomorph**: Unknown.

**Culture characteristics.** Colonies on PDA slow-growing, attaining a diameter of 1.2–1.5 cm in 21 d at 25 °C. Stromatic colonies pulvinate, margin white to pale yellow, conidial masses pale yellow.

**Habitat.** On scale insects (Coccidae, Hemiptera) or whiteflies (Aleyrodidae, Homoptera), found on lower leaf surface of dicotyledons.

**Distribution.** China, Hunan Province, Zhuzhou City.

**Additional specimen examined.** China: Hunan Province, Zhuzhou City, Yanling County, Taoyuandong Reserve, 113°56′30″–114°06′20″ E, 26°18′00″–26°35′30″ N, found on abaxial surface of dicot leaves, 5 October 2017, J. Z. Qiu, paratype HMAS 247795; exparatype living culture CGMCC 3.18914.

**Figure 4:**
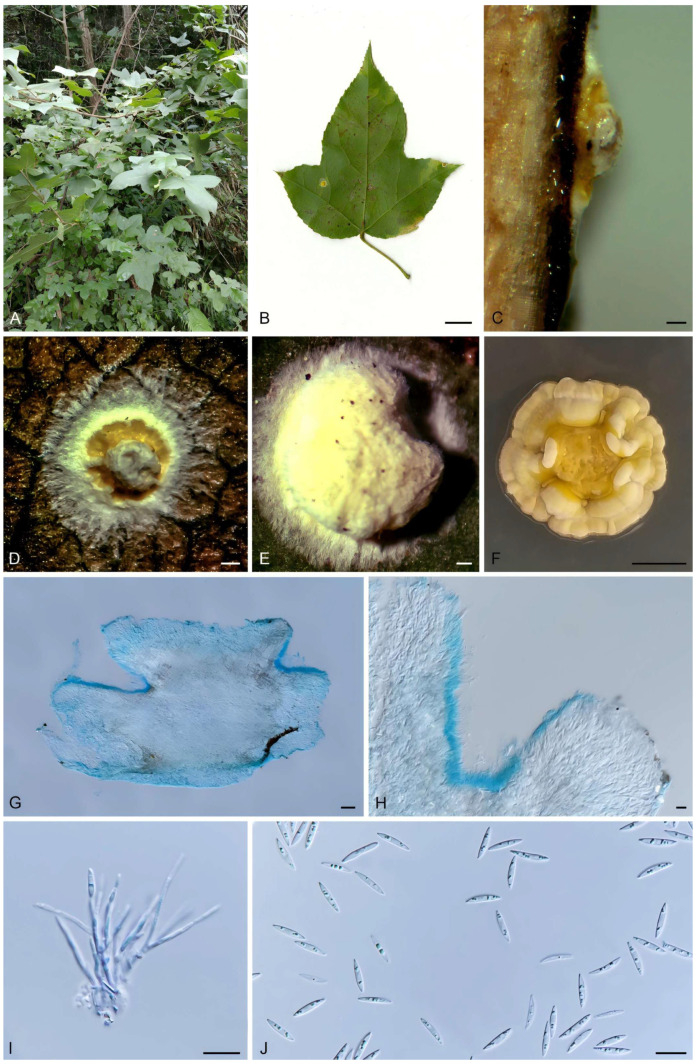
Morphology of *M. microstroma*. (**A**) Habitat; (**B**) stroma on the underside of leaves; (**C**) flank of stroma; (**D**,**E**) frontage of stroma; (**F**) colony on PDA; (**G**,**H**) section of stromata showing conidiomata; (**I**) conidiogenous cells; (**J**) conidia. Scale bars: (**B**) = 1 cm; (**C**–**E**) = 0.2 mm; (**F**) = 0.5 cm; (**G**) = 100 μm; (**H**) = 20 μm; (**I**,**J**) = 10 μm.

**Notes.** In this study, the new species *M. microstroma* was strongly supported (100% ML/1 PP) based on phylogenetic analyses using sequences of three gene regions, and it formed a sister clade to *M. flava* (Figure 1). Morphologically, although the stromatal color and the shape and size of the conidia in the anamorph of *M. microstroma* are similar to those of *M. flava*, stromata of *M. microstroma* (1.18 (0.78–1.64) × 1.03 (0.56–1.59) mm) are slightly smaller than those of *M. flava* (1–4.5 mm). Furthermore, *M. microstroma* is distinguished by the presence of multiple orange acervuli (Figure 4D) and multiple conidiomata (Figure 4G) on each stroma, which contrast markedly with the condition in *M. flava*. Accordingly, this fungus is described here as a new species.

#### 3.4.2. *Moelleriella phukhiaoensis*, Mongkol.,Thanakitp & Luangsa-ard, Fungal Diversity 2016, 78, 1–237 [36] (Figure 5)

**Description.** Stromata pulvinate, yellow, 2.14 (1.23–3.46) × 1.95 (1.19–2.89) mm in diameter and 0.55 (0.30–0.81) mm height; without hypothallus; hyphae compact. Conidiomata numerous, tear-shaped, regularly arranged in the central region of the stroma, 183.79 (103.45–234.48) × 77.95 (55.17–106.9) μm. Conidia fusiform, 11.75 (9.17–13.1) × 1.60 (1.31–2.02) μm, L/W ratio = 7.52.

**Teleomorph**: Unknown.

**Culture characteristics.** Colonies on PDA slow-growing, attaining a diameter of 1.5 cm in 21 d at 25 °C. Hyphae are pale yellow, and abundant yellow conidial masses are visible on the colony surface.

**Specimen examined.** China: Hunan Province, Zhuzhou City, Yanling County, Taoyuandong Reserve, 113°56′30″–114°06′20″ E, 26°18′00″–26°35′30″ N; on underside of dicotyledonous leaves, 15 October 2017, J. Z. Qiu (paratype: HMAS 247937; exparatype living culture: CGMCC 3.19091). China: Jiangxi Province, Longnan County, Jiulian Mountain Nature Reserve, 114°30′–114°50′ E, 24°30′–24°50′ N, 23 September 2017, J. Z. Qiu (paratype: HMAS 247786).

**Notes.** *Moelleriella phukhiaoensis* was originally found on the underside of dicotyledonous leaves in Bueng Pan Protect Forest Unit, Phu Khiao Wildlife Sanctuary, Chaiyaphum Province, Thailand. Hosts are scale insect nymphs (*Hemiptera*). In this study, two specimens, HMAS 247937 and HMAS 247786, formed a sister clade with *M. phukhiaoensis* (BCC 19769, BCC 19773) in the phylogenetic analysis (Figure 1). Morphologically, both HMAS 247937 and HMAS 247786 share the yellow, stromata flattened pulvinate with the holotype of *M. phukhiaoensis* (BBH 17305, ex-type living culture BCC 19769). The shapes of the conidiomata and conidia of the anamorph are also like those of *M. phukhiaoensis* BBH 17305, although the conidiomata and conidia of the two Chinese collections are shorter. However, the two isolates characterized herein were highly similar to *M. phukhiaoensis* BCC 19769 in LSU (99.88%) and *rpb1* (99.53%). Therefore, we identify our isolates as *M. phukhiaoensis*, marking the first record of *M. phukhiaoensis* in China.

**Figure 5:**
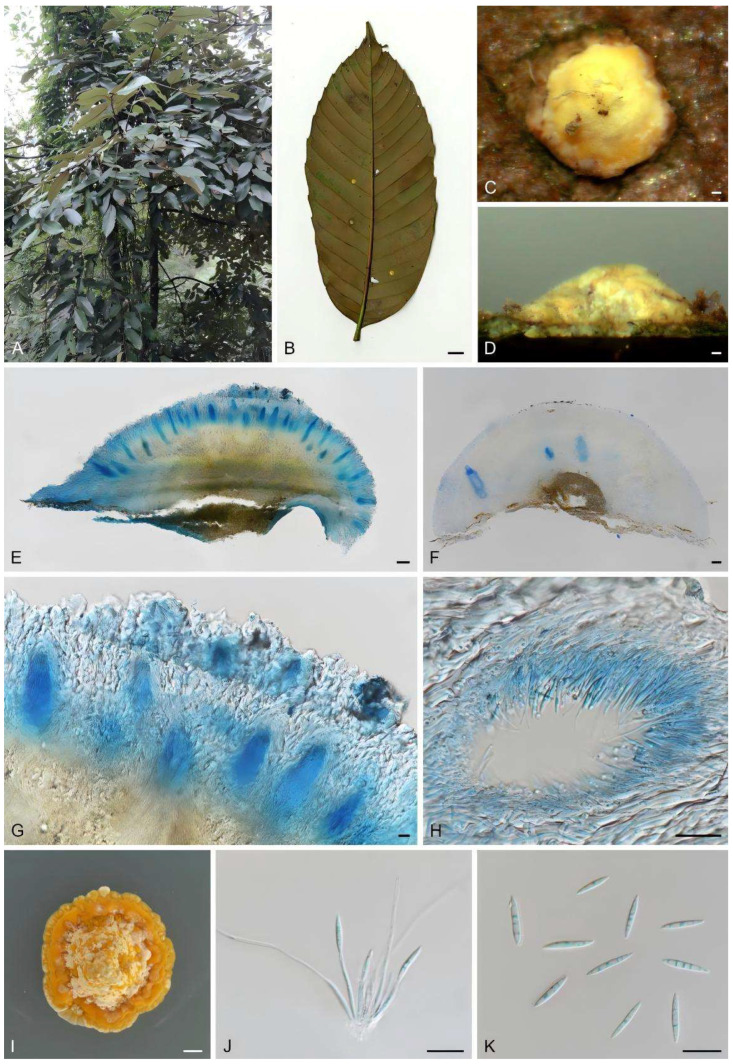
Morphology of *Moelleriella phukhiaoensis*. (**A**) Habitat; (**B**) stroma on the underside of leaves; (**C**) frontage of stroma; (**D**) flank of stroma; (**E**–**H**) section of stromata showing conidiomata; (**I**) colony on PDA; (**J**) conidiogenous cells; (**K**) conidia. Scale bars: (**B**) = 1 cm; (**C**,**D**) = 0.2 mm; (**E**,**F**) = 100 μm; (**G**,**H**) = 20 μm; (**I**) = 0.5 cm; (**J**,**K**) = 10 μm.

#### 3.4.3. *Moelleriella chiangmaiensis*, Khonsanit, A., Noisripoom, W., Mongkolsamrit, S., Phosrithong, N., and Luangsa-ard, J.J., Mycol Progress 2021, 20, 847–867 [3] (Figure 6)

**Description.** Stromata pulvinate, white, 1.66 (0.68–2.96) × 1.40 (0.66–2.03) mm in diameter, 0.36 (0.13–0.61) mm high with a white hypothallus, 0.44 (0.21–0.64) mm wide. The margin of the stromata bears white floccose hyphae, and the surface of mature stromata displays multiple regularly distributed yellow acervuli. Conidiomata are irregular in shape and located at the base of the stromata, 294.87 (208.76–312.82) × 150 (114.67–168.17) µm, containing abundant fusiform conidia 8.53 (6.90–10.10) × 1.83 (1.40–2.20) µm, L/W ratio = 4.75.

**Culture characteristics.** Colonies on PDA fast-growing at 25 °C, attaining 2 cm diameter in 21 d. The mycelium is white, flocose, and loosely textured. Abundant pale yellow conidial masses are observed on the colony surface. Transparent hydrolytic zones, approximately 0.2–0.4 cm in diameter, are present around the colony.

**Specimen examined.** China: Guizhou Province, Libo County, Maolan Reserve, 107°52′10″–108°45′40″ E, 25°09′20″–25°20′50″ N, found on the underside leaves of a dicotyledonous plant, 20 October 2017, J. Z. Qiu, paratype HMAS 247792, HMAS 247793; exparatype living culture CGMCC 3.18915, CGMCC 3.18917.

**Notes:** *Moelleriella chiangmaiensis* was originally discovered on the underside of dicotyledonous leaves in Doi Inthanon National Park, Chiang Mai Province, Thailand. In this study, the specimens identified as *M. chiangmaiensis* (HMAS 247792, HMAS 247793) were also collected from the underside of dicotyledonous leaves. Morphologically, strains CGMCC 3.18915 and CGMCC 3.18917 share with *M. chiangmaiensis* white-to-moderately yellow pulvinate stromata and fusiform conidia of a comparable size (8.53 (6.90–10.10) × 1.83 (1.40–2.20) μm vs. (7–)8–10(–11) × 1.5–2 μm, respectively). Phylogenetic analysis revealed that strains CGMCC 3.18915 and CGMCC 3.18917 formed a monophyletic clade with *M. chiangmaiensis* (BCC 60941, BCC 18029, and BBH 33051) with relatively strong statistical support (99% ML/1 PP, Figure 1). Strains CGMCC 3.18915 and CGMCC 3.18917 exhibit high sequence identity with the ex-type living culture of *M. chiangmaiensis* BCC 18029 in LSU (99.18%) and *tef1-α* (100%). Furthermore, *M. chiangmaiensis* forms a sister clade with *M. puwenensis.* For better resolution, we conducted an additional comparison of strains CGMCC 3.18915 and CGMCC 3.18917 with *M. puwenensis* YHH 2308029. Although the three strains are similar in stromatal morphology and color, strains CGMCC 3.18915 and CGMCC 3.18917 have smaller conidiomata (294.87 (208.76–312.82) × 150 (114.67–168.17) μm than those of *M. puwenensis* (350–545 × 185–250 μm)) and smaller conidia (8.53 (6.90–10.10) × 1.83 (1.40–2.20) μm vs. 10–15 × 1.2–1.9 μm in *M. puwenensis*). On a molecular level, sequence alignment revealed that strains CGMCC 3.18915 and CGMCC 3.18917 show 99.09% sequence identity with *M. puwenensis* YHH 2308029 in the LSU. However, they exhibit lower identity in *rpb1* (98.64%) and *tef1-α* (97.00%), which represents high identity but lower than that seen towards *M. chiangmaiensis.* We therefore identify our isolates as *M. chiangmaiensis*, which represents the first record of this species in China.

**Figure 6:**
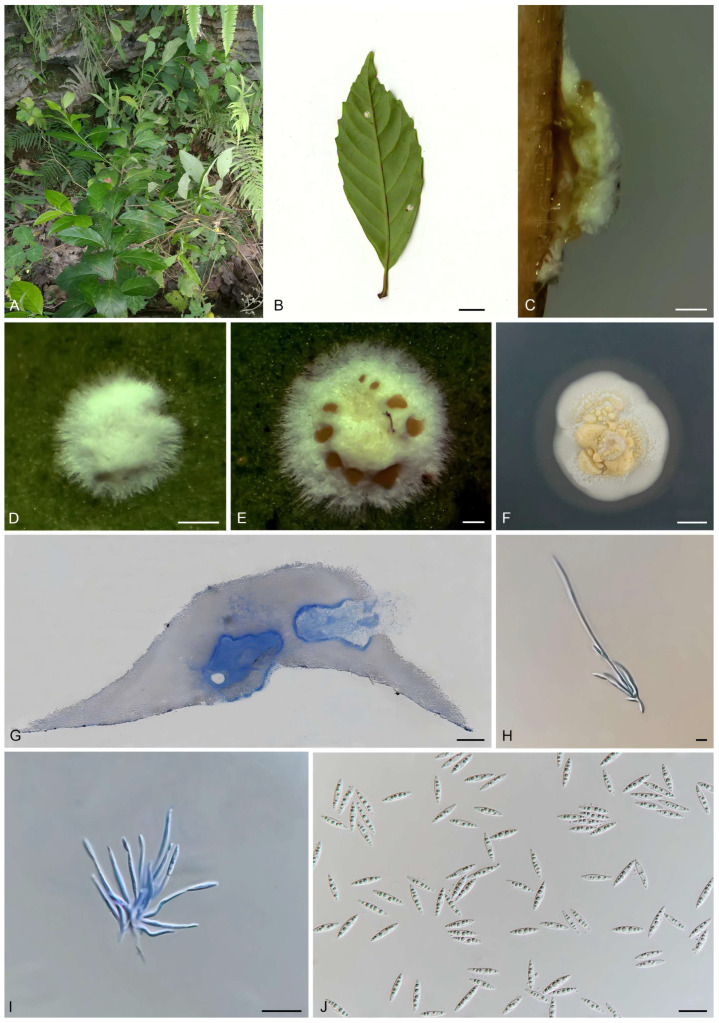
Morphology of *Moelleriella chiangmaiensis*. (**A**) Habitat; (**B**) stroma on the underside of leaves; (**C**) flank of stroma; (**D**,**E**) frontage of stroma; (**F**) colony on PDA; (**G**) section of stromata showing conidiomata; (**H**,**I**) conidiogenous cells; (**J**) conidia. Scale bars: (**B**) = 1 cm; (**C**–**E**) = 0.2 mm; (**F**) = 0.5 cm; (**G**) = 100 μm; (**H**,**I**) = 20 μm; (**J**) = 10 μm.

## 4. Discussion

The genus *Moelleriella* is divided into the effuse and globose clades (A and B, respectively) with the former separated into two sister subclades (I and II) [2,3]. Phylogenetic analyses identified 41 species within Clade A: 19 in Subclade I (including one new species) and 22 in subclade II. Morphologically, members of this clade are characterized by effuse-to-thin, pulvinate stromata that are predominantly cream-colored, occasionally pale yellow to orange [37,38]. Most species have obpyriform or flask-shaped perithecia and fusoid conidia. Clade B encompasses 13 species, which typically exhibit globose, deep yellow to brown stromata and small conidia, occasionally producing large part-spores [3,9]. However, none of these traits are exclusive to either clade. In recent years, 23 new *Moelleriella* species have been reported: 18 from China and five from Thailand [3,9,14]. In the present study, specimens collected from Guizhou, Hunan, and Jiangxi provinces in China were examined morphologically and all were placed within Subclade I of the effuse (A) clade. These included a new species, *Moelleriella microstroma*, which differed significantly from its closest relative, *M. flava*, in both morphology and DNA sequence data. Additionally, two species previously reported in Thailand, *M. chiangmaiensis* and *M. phukhiaoensis*, are reported and illustrated here in China.

In recent years, molecular dating methods that combine analyses of fungal fossils with characterization of selected molecular loci have been widely applied to estimate the divergence times of fungi, significantly enhancing our understanding of fungal evolution at the taxonomic level [39,40,41]. Ancestral state reconstruction and divergence time estimation have inferred that Hypocrealean fungi likely existed in the Early Jurassic [31], with the crown node age estimated at ~193 Mya (158–232 Mya). These analyses suggested that the familial lineages within this group primarily originated in the Late Jurassic and underwent diversification during the Cretaceous. The crown age of Clavicipitaceae was estimated to be at least ~117 Mya (95–144 Mya), dating back to the Early Cretaceous. Additional estimations of the crown age of Hypocreales are believed to be around 200 Mya (174–232 Mya), with the crown node age of Clavicipitaceae refined to 107 Mya (90–126 Mya) [35]. The latter study also provided evidence that the direction of host shifts in Hypocrealean fungi occurred from plants (endophytes) to fungi (pathogens), and then possibly to animals (pathogens). The Cretaceous diversification and cladogenesis within this family gave rise to several subclades, reflecting multiple inter-kingdom host shifts among the three major eukaryotic kingdoms—animals, plants, and fungi [42]. Based on divergence time analysis, our analyses estimate the mean crown age of Clavicipitaceae at ~102.00 Mya (101.00–103.91 Mya), consistent with previous studies. The mean crown age of *Moelleriella* was estimated at ~81.63 Mya (68.88–92.68 Mya), suggesting a Late Cretaceous origin for its ancestor with most species emerging during the Neogene. Furthermore, the mean crown ages of the effuse clade and the globose clade within *Moelleriella* were similar, estimated at ~69.73 Mya and ~70.51 Mya, respectively, suggesting a critical adaptive branching occurred within that timeframe.

Based on our biogeographic analyses, Asia is inferred to be the most probable ancestral region of *Moelleriella*, with Southeast Asia likely representing the center of origin. The now-submerged Bering land bridge (BLB) may have facilitated the dispersal of *Moelleriella* from East Asia to North America [43]. Further analysis indicates that by the Early Oligocene (approximately 33.9 Mya), *M. disjuncta*, *M. epiphylla*, and *M. turbinata* already occurred in both North and South America. Although a direct land bridge between North and South America did not exist during this period, *Moelleriella* may have dispersed via transoceanic or long-distance dispersal (LDD) [44]. Although such latter mechanisms are generally considered rare, this hypothesis may better explain the distribution patterns of fungi across various contexts and geographical regions. Additionally, three species of *Moelleriella* (*M. africana*, *M. mollii*, and *M. raciborskii*) were present in Africa during the Neogene (23.03–2.58 Mya). The collision of the Arabian Plate with the Eurasian Continent around 15 Mya formed a land bridge, which may have facilitated the dispersal of *Moelleriella* species from Asia into Africa via this Arabian Peninsula land bridge [45,46]. However, it is also possible that our dataset may have some (locational) sampling bias, and that additional collection worldwide would alter some of the biogeographic analyses. Overall, our data provide new insights into the diversity, ecology, and evolution of *Moelleriella*, suggesting pathways for species dispersal, vicariance, and expansion that may be associated with the emergence of land bridges as well as long-distance dispersal. Additional sampling and coverage across biogeographic areas combined with additional fossil evidence would help support and/or revise these findings. As these fungi are insect pathogens characterized by brightly colored stromata and tend to occur in tropical/subtropical old-growth forests, where they have specialized to infect scales and whiteflies (causing epizootic infections), they likely help control populations of these insects in forests and may therefore serve as important novel biological control agents.

## 5. Conclusions

Based on the combination of morphological observations and phylogenetic analyses, we identified *Moelleriella* species collected from three provinces in China, recognizing one new species and two new records of species. Simultaneously, divergence time molecular-clock analyses coupled with ancestral state reconstruction (RASP) infers that the genus *Moelleriella* originated in Asia during the Late Cretaceous, approximately 91.60 Mya, and subsequently dispersed to North America, South America, and Africa. This study enriches our understanding of the diversity of *Moelleriella* species and provides a theoretical framework for elucidating the origin and evolution of the genus.

## Figures and Tables

**Table 1:** PCR primers and their annealing temperatures used in this study.

Gene	Primers Name	Sequence (5′-3′)	Annealing Temperature
LSU	LR0Rf	GTACCCGCTGAACTTAAGC	50 °C [17,18]
	LR5r	ATCCTGAGGGAAACTTC	
*tef1* *-α*	EF1α-983f	GCYCCYGGHCAYCGTGAYTTYAT	55 °C [19,20]
	EF1α-2218r	ATGACACCRACRGCRACRGTYTG	
*rpb1*	RPB1-Af	CAYCCWGGYTTYATCAAGAA	50 °C [19,20,21]
	RPB1-Cr	CCNGCDATNTCRTTRTCCATRTA	

**Table 2:** List of species and GenBank accession numbers of sequences used in this study.

Species	Strain	Origin	GenBank Accession no.				
			LSU	SSU	*tef1-α*	*rpb1*	*rpb2*
*Aciculosporium take*	MAFF 241224	Japan	LC571753	-	LC572034	-	LC572048
*A. take*	TNS-F-60465	Japan	LC571756	-	LC572035	-	LC572049
*Albacillium fuzhouense*	CGMCC3.27818	China	PQ425618	PQ425616	PQ469143	PQ469145	PQ469147
*A. fuzhouense*	CGMCC3.27815	China	PQ425619	PQ425617	PQ469144	PQ469146	PQ469148
*Aschersonia badia*	BCC 8105	Thailand	DQ518752	DQ522537	DQ522317	DQ522363	DQ522411
*A. placenta*	BCC 7869	Thailand	JN940907	JN940988	-	JN987885	
*Balansia henningsiana*	GAM 16112	USA	AY545727	AY545723	AY489610	AY489643	DQ522413
*Claviceps fusiformis*	ATCC 26019	Zimbabwe	U17402	DQ522539	DQ522320	DQ522366	
*C. purpurea*	S.A. cp11	Germany	EF469075	EF469122	EF469058	EF469087	EF469105
*Conoideocrella fenshuilingensis*	YHH CFFSL2310002	China	PP178583	-	PP776168	PP776158	-
*C. fenshuilingensis*	YHH CFFSL2310003	China	PP178584	-	PP776169	PP776159	-
*Epichloe elymi*	C. Schardl 760	USA	AY986924	-	AY986951	DQ000352	-
*E. typhina*	ATCC 56429	USA	U17396	U32405	AF543777	AY489653	DQ522440
*Helicocollum surathaniensis*	BCC34464	Thailand	KT222329	-	KT222337	-	-
*Heteroepichloe sichuanensis*	SICAUCC 23-0019^T^	China	OR405917	OR405932	OR531518	-	OR531523
*Hymenostilbe aurantiaca*	OSC 128578	USA	DQ518770	DQ522556	DQ522345	DQ522391	DQ522445
*Hypocrella discoidea*	BCC 8237	Thailand	DQ384937	-	DQ384977	DQ385000	DQ452461
*Keithomyces carneus*	CBS 239.32	France	NG_057769	EF468988	EF468789	EF468894	EF468938
*Marquandomyces sinensis*	ZY 22.064	USA	OR680607	-	OR858937	-	OR842958
*Metapochonia bulbillosa*	CBS 145.70	Denmark	AF339542	AF339591	EF468796	-	EF468943
*Metarhizium album*	ARSEF 2082	Indonesia	DQ518775	DQ522560	DQ522352	DQ522398	DQ522452
*M. viridulum*	BCC 36261	Thailand	MN781827	MN781930	MN781680	MN781737	MN781781
*Moelleriella africana*	P.C. 736	Ghana	AY986917	-	AY986943	DQ000344	-
*M. alba*	BCC49409^T^	Thailand	JQ269646	-	KX254423	JQ256906	-
*M. alba*	BCC49492	Thailand	JQ269645	-	KX254424	JQ256905	-
*M. basicystis*	F183147	Panama	EU392577	-	EU392653	-	-
*M. basicystis*	P.C.374	Costa Rica	AY986903	-	AY986928	DQ000329	-
*M. boliviensis*	P.C.603	Bolivia	AY986923	-	AY986950	DQ000351	-
*M. boehmeriae*	SICAUCC 25-0061^T^	China	PV124784	-	PV153478	PV153492	-
*M. boehmeriae*	SICAUCC 25-0062	China	PV124785	-	PV153479	PV153493	-
***M. chiangmaiensis***	**CGMCC 3.18915**	**China**	**PQ877335**	**-**	**PV505213**	**PV505209**	-
***M. chiangmaiensis***	**CGMCC 3.18917**	**China**	**PQ877336**	**-**	**PV505214**	**PV505210**	-
*M. chiangmaiensis*	BCC60941	Thailand	MT659361	-	MT672278	MT672270	-
*M. chiangmaiensis*	BCC18029^T^	Thailand	MT659360	-	MW091560	-	-
*M. chiangmaiensis*	BBH33051	Thailand	MT659362	-	MT672277	MT672269	-
*M. chumphonensis*	BCC47574^T^	Thailand	JQ269647	-	KX254421	JQ256907	-
*M. chumphonensis*	BBC47575	Thailand	JQ269648	-	KX254422	JQ256908	-
*M*. *cinnamomum*	SICAUCC 25-0067^T^	China	PV124788	-	PV153484	PV153494	-
*M*. *cinnamomum*	SICAUCC 25-0068	China	PV124789	-	PV153485	PV153495	-
*M*. *citrus*	SICAUCC 25-0059^T^	China	PV124782	-	PV153476	PV153490	-
*M*. *citrus*	SICAUCC 25-0060	China	PV124783	-	PV153477	PV153491	-
*M. disjuncta*	J.B.205	Panama	EU392578	-	EU392654	-	-
*M. epiphylla*	P.C. 545	Bolivia	EU392585	-	EU392660	EU392711	-
*M. epiphylla*	I93-813	Guyana	EU392583	-	EU392656	EU392707	-
*M. eucalypti*	SICAU 25-0072^T^	China	PV124778		PV153470	PV153486	
*M. eucalypti*	SICAU 25-0073	China	PV124779		PV153471	PV153487	
*M. eucalypti*	SICAU 25-0074	China	PV124780		PV153472	PV153488	
*M. eucalypti*	SICAU 25-0075	China	PV124781		PV153473	PV153489	
*M. evansii*	P.C. 627	Ecuador	AY986916	-	AY986942	DQ000343	-
*M. flava*	BCC60924^T^	Thailand	KF951146	-	KX254430	MT672271	-
*M. flava*	BCC60925	Thailand	KF951147	-	KX254431	MT672272	-
*M. globostromata*	YFCC 22109275^T^	China	OR828408	-	OR831942	OR831952	-
*M. globostromata*	YHH 221010	China	OR828403	-	OR831940	OR831950	-
*M. gracilispora*	CGMCC3.18989	China	KC964202	-	KC964191	KC964179	-
*M. gracilispora*	CGMCC3.18990	China	KC964203	-	KC964192	KC964180	-
*M. hainanensis*	YHH 2303020	China	OR828400	-	OR831938	OR831948	-
*M. hainanensis*	YFCC 23039277^T^	China	-	-	OR831939	OR831949	-
*M. insperata*	ARSEF 2396^T^	Philippines	AY518374	-	DQ070029	EU392713	-
*M. jinghongensis*	YFCC 23089312^T^	China	-	-	OR854253	OR837093	-
*M. jinghongensis*	YHH 2308028	China	OR828410	-	OR854256	OR837096	-
*M. jinuoana*	YHH MJBP2309031^T^	China	PP178643	-	PP177170	PP177160	-
*M. jinuoana*	YHH MJBP2309032	China	PP178644	-	PP177171	PP177161	-
*M. kanchanaburiensis*	BCC75980	Thailand	MT659364	-	MT672280	MT843901	-
*M. kanchanaburiensis*	BCC75981^T^	Thailand	MT659365	-	MT672281	-	-
*M. libera*	P.C. 444	Mexico	EU392591	-	EU392662	EU392714	-
*M. libera*	P.C. 445	Mexico	AY986900	-	AY986925	DQ000326	-
*M. longzhuensis*	YHH MLFSL2310012^T^	China	PP178646	-	PP177173	PP177163	-
*M. longzhuensis*	YHH MLFSL2310013	China	PP178647	-	PP177174	PP177164	-
*M. macrostroma*	J.B. 115	Costa Rica	AY986920	-	AY986947	DQ000348	-
*M. macrostroma*	P.C. 605	Bolivia	AY986919	-	AY986946	DQ000347	-
*M. madidiensis*	P.C. 569	Bolivia	AY986915	-	AY986941	DQ000342	-
*M. madidiensis*	P.C. 594	Bolivia	EU392595	-	EU392666	EU392718	-
***M. microstroma***	**CGMCC 3.18913^T^**	**China**	**PQ877337**	**-**	**PV505215**	**PV505211**	**-**
***M. microstroma***	**CGMCC 3.18914**	**China**	**PQ877338**	**-**	**PV505216**	**PV505212**	**-**
*M. mollii*	I93-901A	Côte D’Ivoire	EU392599	-	EU392667	EU392719	-
*M. mollii*	I93-901C	Côte D’Ivoire	EU392600	-	EU392668	EU392720	-
*M. multiperitheciata*	YFCC 23089307^T^	China	OR828407	-	OR832085	OR837089	-
*M. multiperitheciata*	YFCC 22109308	China	OR828406	-	OR832086	OR837090	-
*M. nanensis*	BCC66303^T^	Thailand	KX298236	-	KX254427	MW085940	-
*M. nanensis*	BCC66305	Thailand	MW080317	-	KX254428	MW085941	-
*M. nivea*	BCC60891^T^	Thailand	MW080318	-	MT672282	MW085942	-
*M. nivea*	BCC58543	Thailand	MT659366	-	MT672283	MT672274	-
*M. ochracea*	IE 1308	Mexico	EU392601	-	EU392669	EU392721	-
*M. ochracea*	P.C.648	Honduras	EU392605	-	EU392671	EU392723	-
*M. oxystoma*	BCC 8406	India	DQ384943	-	DQ384978	DQ384993	-
*M. oxystoma*	BCC 9482	India	DQ377986	-	DQ384957	DQ385013	-
***M. phukhiaoensis***	**HMAS 247937**	**China**	**PV817736**	**-**	**PV832432**	**PV832430**	**-**
***M. phukhiaoensis***	**HMAS 247786**	**China**	**PV817737**	**-**	**PV868264**	**PV832431**	**-**
*M. phukhiaoensis*	BCC19769	Thailand	KT880502	-	-	KT880506	-
*M. phukhiaoensis*	BCC19773	Thailand	KT880503	-	-	KT880507	-
*M. phyllogena*	P.C. 555	Bolivia	EU392610	-	EU392674	EU392726	-
*M. phyllogena*	J.B. 130	Panama	EU392608	-	EU392672	EU392724	-
*M. pongdueatensis*	BCC31787^T^	Thailand	KT880500	-	KX254433	KT880504	-
*M. pongdueatensis*	BCC31788	Thailand	KT880501	-	KX254434	KT880505	-
*M. pseudothanathonensis*	YFCC 22099302^T^	China	-	-	OR842379	OR837103	-
*M. pseudothanathonensis*	YFCC 22099303	China	-	-	OR842380	OR837104	-
*M. puerensis*	YFCC 8615^T^	China	MW786748	-	MW815596	MW815595	-
*M. puerensis*	YFCC 8626	China	MW786750	-	MW815598	MW815594	-
*M. puertoricoensis*	BCC 88320	Puerto Rico	MN954683	-	MN944389	-	-
*M. puertoricoensis*	BCC 88322	Puerto Rico	MN954682	-	MN944391	-	-
*M. pumatensis*	BCC 41004	Vietnam	-	-	HQ722026	-	-
*M. pumatensis*	BCC 41006	Vietnam	-	-	HQ722027	-	-
*M. puwenensis*	YHH 2308029^T^	China	OR828412	-	OR854257	OR831953	-
*M. puwenensis*	YHH 2308031	China	OR828414	-	OR854259	OR831955	-
*M. qionzhongensis*	YHH 2303021	China	OR828399	-	OR831936	OR831946	-
*M. qionzhongensis*	YFCC 23039306^T^	China	-	-	OR831937	OR831947	-
*M. raciborskii*	AFR28	Ghana	DQ070113	-	EU392675	EU392727	-
*M. raciborskii*	I93-901	Côte D’Ivoire	EU392611	-	EU392676	EU392728	-
*M. reineckeana*	BCC1713	Thailand	-	-	DQ384968	DQ385008	-
*M. reineckeana*	BCC1765	Thailand	-	-	DQ384958	DQ385010	-
*M. rhombispora*	P.C. 467	Costa Rica	AY986908	-	AY986933	DQ000334	-
*M. rhombispora*	P.C. 696	Honduras	EU392618	-	EU392680	EU392732	-
*M. schizostachyi*	CBS 100067	Thailand	AY986921	-	AY986948	DQ000349	-
*M. simaoensis*	YHH 2210015^T^	China	OQ621807	-	OQ623179	OQ616915	-
*M. simaoensis*	YHH 2210016	China	OQ621808	-	OQ623180	OQ616916	-
*M. sinensis*	CGMCC 3.18911	China	MK412091	-	-	MK412101	-
*M. sinensis*	BCC60932	Thailand	MT659368	-	-	MT672275	-
*M. sloaneae*	I94-920	Guatemala	EU392621	-	EU392682	EU392734	-
*M. sloaneae*	I94-922C	Belize	EU392622	-	EU392683	EU392735	-
*M. thanathonensis*	MFLU:16-2922	Thailand	-	-	KY646200	-	-
*M. turbinata*	IMI 352838	Mexico	EU392625	-	EU392685	EU392737	-
*M. turbinata*	P.C. 678	Honduras	EU392627	-	EU392687	EU392739	-
*M. umbospora*	P.C. 461	Mexico	EU392628	-	EU392688	EU392740	-
*M. umbospora*	P.C. 457	Mexico	AY986904	-	AY986929	DQ000330	-
*M. yuanyangensis*	YFCC 23039314^T^	China	OR828405	-	OR831945	OR837097	-
*M. yuanyangensis*	YHH 2209001	China	-	-	OR831944	OR837098	-
*M. yunnanensis*	YFCC 23089310^T^	China	-	-	OR832093	OR837102	-
*M. yunnanensis*	YHH 2308001	China	OR828416	-	OR832091	OR837100	-
*M. zhongdongii*	P.C. 504	Costa Rica	EU392631	-	EU392689	EU392741	-
*M. zhongdongii*	P.C. 549	Bolivia	EU392632	-	EU392690	EU392742	-
*Myriogenospora atramentosa*	A.E.G. 96–32	Cuba	AY489733	AY489701	AY489628	AY489665	DQ522455
*Neoaraneomyces araneicola*	DY101711	China	MW730609	-	MW753033	MW753024	MW753026
*N. wuyishanensis*	CGMCC3.28307	China	PQ278803	PQ286042	PQ301444	PQ316536	PQ334680
*N. wuyishanensis*	CGMCC3.28308	China	PQ278804	PQ286043	PQ301445	PQ316537	PQ334681
*Nigelia aurantiaca*	BCC 13019	Thailand	GU979948	GU979939	GU979957	GU979966	GU979971
*Ophiocordyceps brunneipunctata*	OSC 128576	USA	DQ518756	DQ522542	DQ522324	DQ522369	DQ522420
*O. entomorrhiza*	KEW 53484	USA	EF468809	EF468954	EF468749	EF468857	EF468911
*O. gracilis*	EFCC 3101	USA	EF468810	EF468955	EF468750	EF468858	EF468913
*O. irangiensis*	OSC 128577	USA	DQ518760	DQ522546	DQ522329	DQ522374	DQ522427
*O. nutans*	OSC 110994	USA	DQ518763	DQ522549	DQ522333	DQ522378	-
*O. sobolifera*	KEW 78842	USA	EF468828	EF468972	-	EF468875	EF468925
*O. sphecocephala*	OSC 110998	USA	DQ518765	DQ522551	DQ522336	DQ522381	DQ522432
*O. nigrella*	EFCC 9247	USA	EF468818	EF468963	EF468758	EF468866	EF468920
*Orbiocrella petchii*	NHJ 6240	Thailand	EU369038	EU369103	EU369022	EU369060	EU369082
*O. petchii*	NHJ 6209	Thailand	EU369039	EU369104	EU369023	EU369061	EU369081
*Papiliomyces albastromata*	YHH 2307002	China	OR770504	OR770494	PP479838	PP203269	PP479841
*P. albastromata*	YHH 2307003	China	OR770503	OR770493	PP479837	PP203268	PP479840
*Parametarhizium changbaiense*	SGSF125	China	MN589994	MN59023	MN908589	MN917168	MT921829
*Parametarhizium hingganense*	SGSF355	China	MN061635	-	MN065770	MN917170	-
*Paraneoaraneomyces sinensis*	ZY 22.006	China	OQ709260	OQ709248	OQ719626	-	OQ719621
*P.s sinensis*	ZY 22.007	China	OQ709261	OQ709249	OQ719627	-	OQ719622
*Pochonia boninensis*	JCM 18597	Japan	AB709831	AB758255	AB758463	AB758666	AB758693
*Purpureocillium lilacinum*	CBS 284.36	USA	-	AY526475	EF468792	EF468898	EF468941
*P. maesotensis*	BCC 88441	Thailand	MN781877	-	MN781734	MN781779	MN781824
*Samuelsia chalalensis*	P.C. 560	Bolivia	EU392637	-	EU392691	EU392743	-
*S. mundiveteris*	BCC40021	Thailand	GU552152	-	GU552145	-	-
*S. mundiveteris*	BCC40022	Thailand	GU552153	-	GU552146	-	-
*S. rufobrunnea*	P.C. 613	Bolivia	AY986918	-	AY986944	DQ000345	-
*Shimizuomyces paradoxus*	EFCC 6279	Korea	EF469084	EF469131	EF469071	EF469100	EF469117
*S. paradoxus*	EFCC 6564	Korea	EF469083	EF469130	EF469072	EF469101	EF469118
*Yosiokobayasia kusanagiensis*	TNS-F 18494	Japan	JF415972	JF415954	JF416014	JN049890	-

Note: Newly generated sequences are in bold. The ex-type, ex-epitype, ex-neotype strains are marked with “^T^”.

## Data Availability

Data are available at NCBI (https://www.ncbi.nlm.nih.gov/, accessed on 28 January 2026).
